# Cohort Profile: The Nor-Work Cohort

**DOI:** 10.1093/ije/dyaf019

**Published:** 2025-03-04

**Authors:** Rachel L Hasting, Suzanne L Merkus, Karina Undem, Jorunn Kirkeleit, Rune Hoff, Jon Michael Gran, Karl-Christian Nordby, Ingrid S Mehlum

**Affiliations:** Research Group for Occupational Medicine and Epidemiology, National Institute of Occupational Health, Oslo, Norway; Research Group for Work Psychology and Physiology, National Institute of Occupational Health, Oslo, Norway; Research Group for Occupational Medicine and Epidemiology, National Institute of Occupational Health, Oslo, Norway; Research Group for Occupational Medicine and Epidemiology, National Institute of Occupational Health, Oslo, Norway; Research Group for Occupational Medicine and Epidemiology, National Institute of Occupational Health, Oslo, Norway; Oslo Centre for Biostatistics and Epidemiology, Oslo University Hospital, Oslo, Norway; Department of Biostatistics, Oslo Centre for Biostatistics and Epidemiology, University of Oslo, Oslo, Norway; Research Group for Occupational Medicine and Epidemiology, National Institute of Occupational Health, Oslo, Norway; Research Group for Occupational Medicine and Epidemiology, National Institute of Occupational Health, Oslo, Norway; Institute of Health and Society, University of Oslo, Oslo, Norway; Department of Occupational and Environmental Medicine, Copenhagen University Hospital—Bispebjerg and Frederiksberg, Copenhagen, Denmark; Department of Public Health, University of Copenhagen, Copenhagen, Denmark

**Keywords:** cohort profile, occupational health, occupational exposure, working life

Key Features

- The Nor-Work cohort was established in 2018 to identify differences in health, labour force participation, and related outcomes, and to study how these outcomes are associated with other factors, including occupation and occupational exposures. The dynamic cohort consists of individuals born between 1930 and 1992 who have ever lived and/or worked in Norway and who have a Norwegian personal identification number (*N* = 5 799 138). Data are available from 1960 to 2022, though exact time periods vary between data sources.
- Nor-Work contains data from several national administrative registers, which allows for prospective follow-up. The register-based nature means individuals cannot opt out, and thus there is no attrition. The cohort is mainly intended for occupational epidemiological research, and contains information on employment, occupation and industry, diagnoses from specialist healthcare and health registries, health-related benefits and their diagnoses (sickness absence, disability benefits), as well as demographic and socioeconomic factors.
- Occupation is registered from 2003; prior to this, self-reported occupation is available from the censuses in 1960, 1970, and 1980, and for a subset of the population in 1990. For the years in which occupational data are missing, imputed occupation is available.
- Researchers interested in data access or collaboration should contact Karl-Christian Nordby (kcn@stami.no).

## Why was the cohort set up?

Though paid employment can be beneficial to health, aspects of the work environment may also contribute to the onset and worsening of health issues [1]. Occupational exposures can increase the risk of musculoskeletal and mental disorders, cancer, and respiratory diseases, among others [2, 3]. These in turn can lead to premature withdrawal from paid employment, either temporarily due to sickness absence or unemployment, or permanently through disability and early retirement [4, 5]. The social and financial implications of work-related illness and withdrawal from the workforce (see Box 1) impact the individual, the employer, and society [9, 10]. This highlights the importance of maintaining a healthy population and lengthening employees’ working lives, especially in light of the ageing population [11].

Box 1. A note on employment in Norway

The labour force refers to all who are employed or unemployed (i.e. actively seeking employment), whilst the workforce refers to those who are employed [those aged 15 and over who report (e.g. in the Labour Force Survey) that they have worked in gainful employment for at least one hour in the previous week or who had a job but were absent from work during the reference week] [6]. In Norway, the standard retirement age is 67 [7]. Statistics Norway includes the age range of 15–74 years in their employment statistics [8].

The relationships between various occupational exposures, health, labour force participation (see Box 1), sociodemographic factors, and public policies are complex and dynamic in nature. To account for this in occupational health research, it is important to go beyond simple exposure-outcome associations and adopt a more holistic working life-course perspective [12]. This perspective allows us to quantify how changes in occupational exposures affect dynamic trajectories of health and labour force participation over time and to identify individual and work-related factors that influence or correlate with these trajectories [13, 14]. A working life-course perspective also allows us to assess the effects of targeted interventions on health and labour force participation [7, 14]. This knowledge could help support workers in extending their working life and prevent the development of work-related morbidity and premature death.

The Nor-Work cohort was established in 2018 at the National Institute of Occupational Health (STAMI) in Norway. The purpose of the cohort is to utilize rich national register data to investigate these complex relationships. Due to the register-based nature of the cohort, informed consent is not required, in accordance with the General Data Protection Regulation Articles 6.1 (e) and 9.2 (j) [15]. The cohort was originally funded through a NordForsk project that ran from 2016 to 2019 [16], and has since been extended and applied in several projects examining working conditions, work participation, and work-related health.

## Who is in the cohort?

Nor-Work is a dynamic cohort comprised of individuals born between 1930 and 1992 who have ever lived and/or worked in Norway and are registered with a Norwegian personal identification number (PIN, temporary or permanent; *N* = 5 799 138). The PIN enables the linkage of data from various national registers at an individual level. Companies are also given a unique identification number, which enables linkage of company-specific information (e.g. industry, sector, company size) to individual level data through employment records. Individuals are included either from birth (Norway-born) or from the first date of immigration or registered employment in Norway, and are followed until death or until end of follow-up in the registries, as long as they are residing or working in Norway. Individuals may immigrate and emigrate multiple times. Table 1 shows demographic information for the cohort.

**Table 1.** Demographic information for the Nor-Work cohort (*N* = 5 799 138).

Demographic information	Men (*n* = 3 292 017, 57%)	Women (*n* = 2 507 121, 43%)	% Men
Birth year (quartiles)	1954–1968–1981	1952–1967–1980	n/a
Country group of birth	*n* (%)	*n* (%)	
Norway	1 811 555 (55)	1 716 929 (68)	51
Nordic countries except Norway	115 770 (4)	117 277 (5)	50
Other EU/EEA countries	225 023 (7)	138 847 (6)	62
Europe excluding EU/EEA	79 075 (2)	69 990 (3)	53
Asia, Africa, Latin America, Oceania (except Australia and New Zealand)	194 411 (6)	208 498 (8)	48
USA, Canada, Australia, New Zealand	33 891 (1)	32 506 (1)	51
Unknown	832 292 (25)	223 074 (9)	79
Highest education level			
Lower secondary or below	493 945 (15)	457 043 (18)	52
Upper secondary, basic	270 580 (8)	323 517 (13)	46
Upper secondary, completed	699 162 (21)	442 177 (18)	61
Tertiary, undergraduate	416 558 (13)	583 793 (23)	42
Tertiary, graduate	329 229 (10)	286 271 (11)	53
Missing^a^	1 082 543 (33)	414 320 (17)	72
Ever been employed in Norway (Norway-born only)^b^	1 765 331 (97)	1 675 871 (98)	51
Ever been employed in Norway (foreign-born only)^b^	1 043 484 (70)	591 196 (75)	64
Ever registered as residing in Norway (foreign-born only)^c^	1 302 595 (88)	739 306 (94)	64

EU, European Union; EEA, European Economic Area (Iceland, Liechtenstein, and Norway, in addition to EU).

a Information on education is largely missing for immigrants who have not taken any formal education in Norway.

b Data presented for the following years: 1960, 1970, 1980, 1983–2020.

c Data presented for the following years: 1960–2021.

## How often have they been followed up?

Data are available from 1960 to 2022 (with the exception of sociodemographic information through the National Population Register), with most data recorded either regularly (yearly) or on an event-time basis, meaning we potentially have exact dates for the events. However, this is dependent on both the data source and whether individuals resided entirely in Norway or immigrated/emigrated (see the specific descriptions of data sources for more information). Descriptions of the exact time periods for data can be found in Table 2 and in the description of what has been measured below.

**Table 2.** Key categories and sub-groups in the Nor-Work cohort, as well as source, measurement level, and available time periods (note that this list is not exhaustive)

Key categories	Source	Sub-groups	Measurement level	Time period
Individual/demographic factors	SSB	Gender	Legal gender per 01.01.2022	1930–1992
		Birth	Month/year	1930–1992
		Death	Month/year	1960–2020
		Civil status	Day/month/year; nine categories^a^	1975–2021
		Income (tax-free transfers, some benefits that are tax-exempt)	Yearly; Norwegian kroner (NOK)	1993–2020
		Income (taxable income)	Yearly; Norwegian kroner (NOK)	1967–2020
		Region/municipality of residence	Day/month/year; 4-digit codes^a^	1960, 1970–2021
		Education	Month/Year (highest education level, measured yearly); 6-digit Norwegian Standard Classification of Education codes^a^	1970–2021
Immigration/emigration	SSB	Date of immigration	Day/month/year	1930–2021
		Date of emigration	Day/month/year	1930–2021
		Reason for immigration	Seven categories^a^	1930–2021
		Citizenship	Country^a^	1930–2021
		Country of birth	Country^a^	1930–2021
Labour force participation	SSB	Occupation	NYK65 codes^b^ (3-digit) STYRK-98 codes^a^^,^^c^ (4-digit) STYRK-08 codes^a^^,^^c^ (4-digit)	1960, 1970, 1980, 1990 2003–2021 2009–2021
		Industry^d^	SN-78 codes (3-digit) SN-83 codes (3-digit) SN-94 codes (5-digit) ISIC Rev.3 (5-digit) NACE Rev. 1 & 2 (5-digit) SN-02 codes (5-digit) SN-07 codes (5-digit)	1960, 1970, 1980 1990 1983–1994; 2000–2002 1992–2003 1995–2014 2003–2007 2008–2022
		Employment, event data	Day/month/year (start/stop), number of hours worked	1983–Mar 2022
	Norwegian Welfare and Labour Administration	Norwegian Agreement on a More Inclusive Working Life (IA Agreement)	IA Agreement status (yes/no) & year Amount received in IA grants, Norwegian krone (NOK)	2010–2019 2010–2019
Health outcomes	Cancer Registry of Norway	Cancer data	Diagnosis month/year, certainty, morphology (ICD-O-2/ICD-O-3^e^), location (ICD-7/ICD-10^f^)	1960–2020
	Norwegian Patient Registry	Contact with specialist health services	Year of contact, diagnoses (ICD-10 codes^f^)	2008–2024
	Norwegian Welfare and Labour Administration	Sickness absence/follow-on benefit diagnoses	ICPC-2 codes^g^	1989–2022
		Disability pension diagnoses	ICD-9 codes^f^ ICD-10 codes^f^	1967–2022 1992–2022
	Cause of Death Registry	Causes of death	ICD-10 codes^f^	1960–2022
Health-related and social benefits	SSB	Sickness absence/follow-on benefits	Day/month/year (start/stop), grade	1992–2021
		Disability pension	Month/year (start/stop), grade	1967–2020
		Unemployment benefit	Day/month/year (start/stop)	1992–Mar 2022
		(Early) retirement	Month/year (start), grade	1967–1990, 1994–2020

SSB, Statistics Norway.

a Statistical Classifications and Codelists, Statistics Norway: https://www.ssb.no/en/klass/.

b Nordic Classification of Occupations (NYK) 1965, based on the International Standard Classification of Occupations (ISCO) 1958.

c STYRK is the Norwegian classification based on ISCO; STYRK-98 is based on ISCO-88.

d SN is the Norwegian classification, and was based on the International Standard Industrial Classification of All Economic Activities (ISIC) [17] until SN-94, when it began to be based on the EU Nomenclature of Economic Activities (NACE) Rev. 1 & 2 [18].

e International Classification of Diseases for Oncology (ICD-O), versions 2 and 3 [19].

f International Classification of Diseases (ICD), 10th edition [20].

g International Classification of Primary Care (ICPC) [21].

## What has been measured?

### Data sources

**Figure 1.**
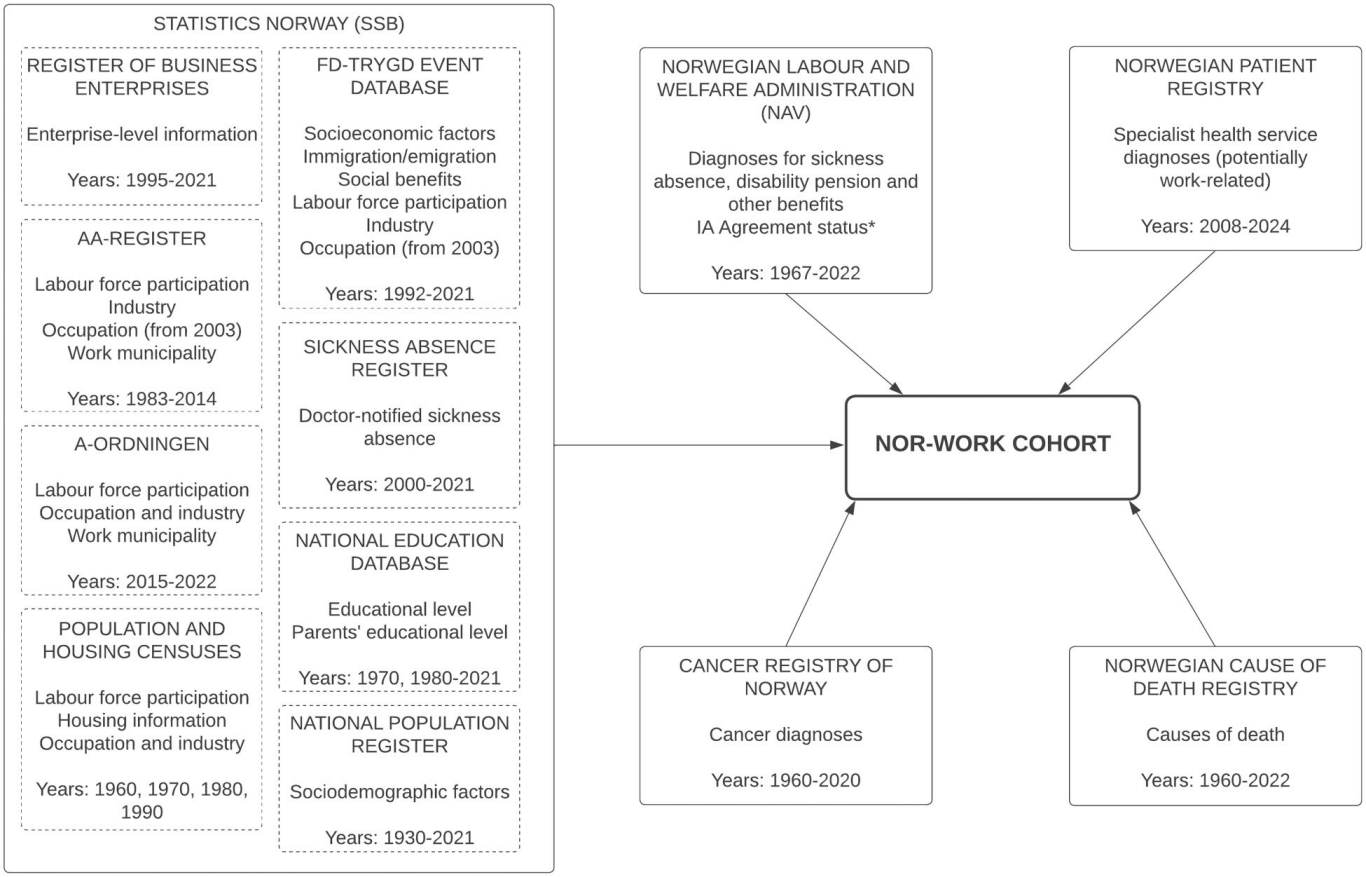
Overview over the sources of the key variable categories in the Nor-Work cohort. * denotes whether companies had signed the national agreement on a more inclusive working life (the IA agreement).

Data come from mandatory national databases, held by Statistics Norway (SSB), the Norwegian Labour and Welfare Administration, the Cancer Registry of Norway, the Norwegian Cause of Death Registry, and the Norwegian Patient Registry (Fig. 1). A summary of the main categories of data, with sub-groups and time periods, is found in Table 2. Note that this list is not exhaustive.

### Sociodemographic information

Sociodemographic variables were obtained from SSB (Fig. 1). Individual and demographic information available in the Nor-Work cohort includes, but is not limited to, birth year and month, gender (legal, not sex), civil status, educational attainment, country of birth, emigration and immigration dates, year and month of death, income, and municipality/region of residence (Table 2).

### Labour force participation and occupational history

The earliest data on employment, occupation, and industry stem from the Population and Housing Censuses [22] and are available for the years 1960, 1970, and 1980, and for a subset of the cohort in 1990 [about 62%, ranging from 9% in the largest municipality (Oslo) to 100% in the smallest municipalities]. From 1983 onwards, information on labour force participation was obtained from SSB and comes from various sources, including the State Register of Employers and Employees (Aa-register), the ‘a-ordning’ scheme [23, 24] and the event database FD-Trygd [25] (Fig. 1). Labour force participation data include information on employment episodes (start and stop date), episodes temporarily out of employment (including unemployment and parental leave), industry, sector (private vs public), and occupation. Some labour force participation information, including occupation, is complete only for employees and not for self-employed individuals; this is due to differences in how self-employed individuals report employment information to the authorities [23]. Additional information, such as working hours and work municipality, are also available for most of the follow-up period. Some company-level information comes from the Central Register of Establishments and Enterprises (Fig. 1). This includes number of employees, industry, sector, and plans to merge with other companies or cease operations [26]. Yearly data on whether companies had joined the Agreement for a More Inclusive Working Life (the IA Agreement) and whether they received grants from the program are available between 2010 and 2019 (following this, all companies gained access to IA measures; for more information on the IA Agreement see [27]).

**Figure 2.**
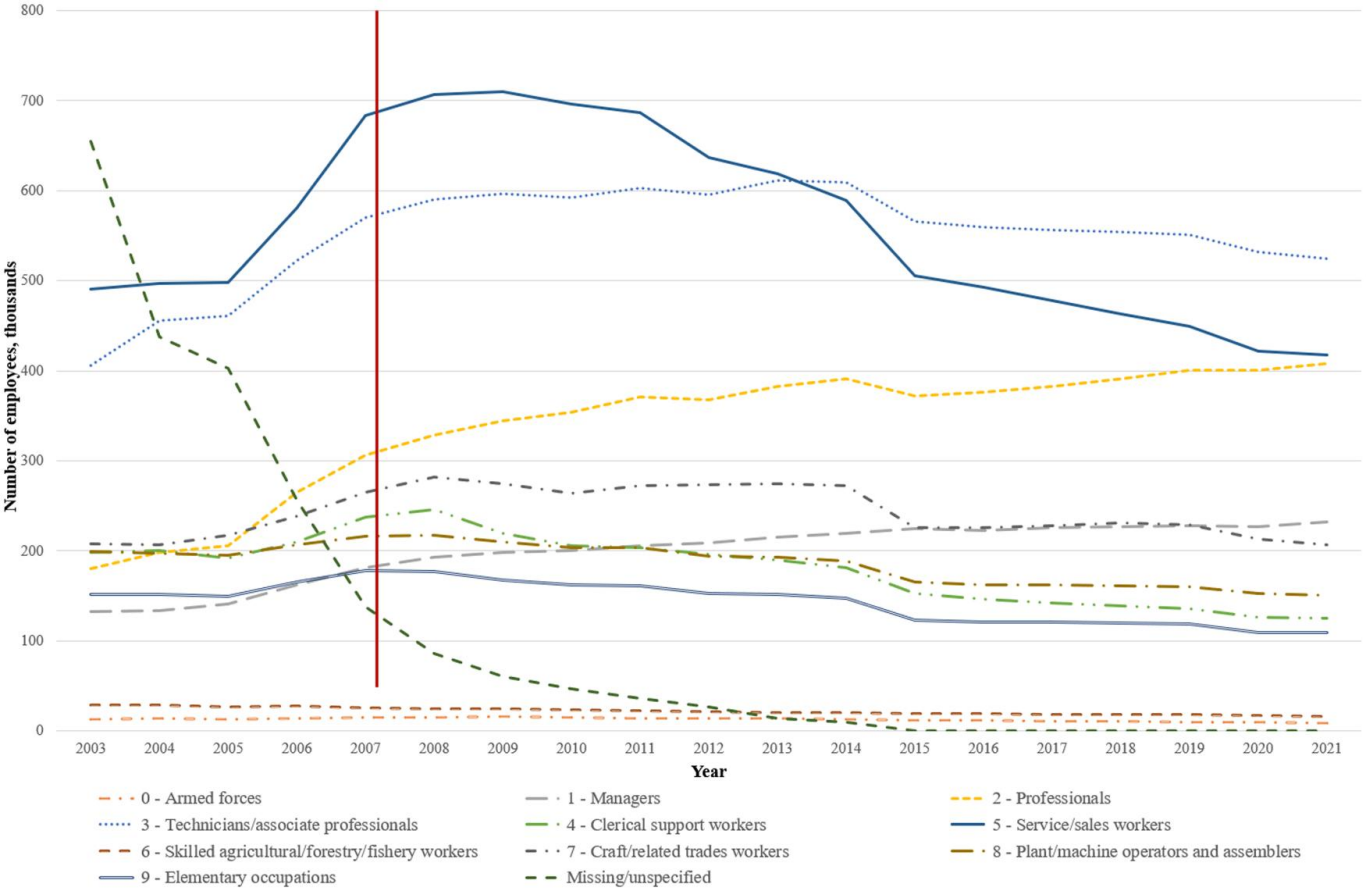
The number of employees in each major occupational group, registered using the Norwegian classification of occupations (STYRK-98, based on the International Standard Classification of Occupations (ISCO)), from 2003 to 2021. The line drawn at 2007 indicates the point at which occupational data were recorded for all employees, including those in the public sector.

Industry is coded according to the industrial classification codes ISIC (International Standard Industrial Classification) or NACE (Classification of Economic Activities in the European Union) [17, 18]. Self-reported occupation is available in the Population and Housing Censuses (described above). Since 2003, companies have reported occupational codes for employment episodes, mainly in the private sector. This practice was gradually expanded to include all employment episodes, encompassing those in the public sector as well. Different occupational codes have been used throughout the different time periods; please refer to Table 2 for more information. Crosswalks have been developed to assist with converting national codes to international codes [e.g. from the Nordic Classification of Occupations (NYK) 1965 and the Norwegian classification STYRK 1998, based on the International Standard Classification of Occupations (ISCO), to ISCO-88(COM), and STYRK-08 to ISCO-08]. Figure 2 depicts the number of employees in each occupational group over time, corresponding to the first digit of the Norwegian STYRK-98 codes [28]. For the years in which occupational data are missing, imputed occupation is available. Occupation was imputed annually five years back and forth in time based on self-reported occupation from the censuses. Additionally, occupation was imputed for persons registered with an employment episode using information on employer, industry codes (three digits), and educational codes (four digits).

### Occupational exposures and job exposure matrices (JEMs)

Information on estimated occupational exposures (e.g. biomechanical, psychosocial, physical, chemical) can be obtained through JEMs, and connected to individuals using the occupational codes described above. JEMs are tools that provide an estimate of exposure to a specific agent or stressor on an occupational level, and a wide range of JEMs are available for many agents and stressors, including a Norwegian gender-specific JEM on mechanical and psychosocial work exposures [29] and the EuroJEM, developed within the framework of the Exposome Project for Health and Occupational Research (EPHOR) project [30]. It is also possible to identify individuals likely to have certain employment conditions, like shift work or precarious work, through other variables in Nor-Work, such as employment type (directly employed or employed by a staffing agency) and number of hours worked per week, rather than through a JEM.

### Health outcomes

Information on cancer cases, including diagnosis month and year and cancer type (location, morphology, and histology), is available between 1960 and 2020 and is ascertained by linkage with the Cancer Registry of Norway [31]. The cases are coded according to a modified version of the International Classification of Diseases (ICD-7/ICD-10) and the International Classification of Diseases for Oncology (ICD-O-2/ICD-O-3) [19, 20].

Diagnostic data on potentially work-related diseases and injuries from specialized inpatient and outpatient healthcare come from the Norwegian Patient Registry [32]. The Norwegian Patient Registry has data on hospitalizations and outpatient visits at somatic and psychiatric hospitals from 2008, as well as visits to contracted private specialists from 2009, registered yearly. Diagnoses are coded according to ICD-10 [20]. Diagnoses related to absence from work come from the Norwegian Labour and Welfare Administration and are available from 1989 for sickness absence (registered with exact dates) and follow-on benefits (registered monthly) and from 1967 for disability pension (registered monthly) [33]. Diagnoses for sickness absence and follow-on benefits are coded according to The International Classification of Primary Care 2 [21], whilst disability pension diagnoses are coded according to ICD-9 or ICD-10 [20].

Data on causes of death (with month and year of death) were obtained from the Norwegian Cause of Death Registry, owned by the Norwegian Institute of Public Health, for the time period 1960–2022 [34]. Causes of death are coded according to the ICD codes (for an overview of which ICD versions were used when, see Norwegian Institute of Public Health [34]) and grouped based on the European Shortlist for causes of death [35], as well as specific diagnoses that are possibly work-related.

### Health-related and social benefits

**Figure 3.**
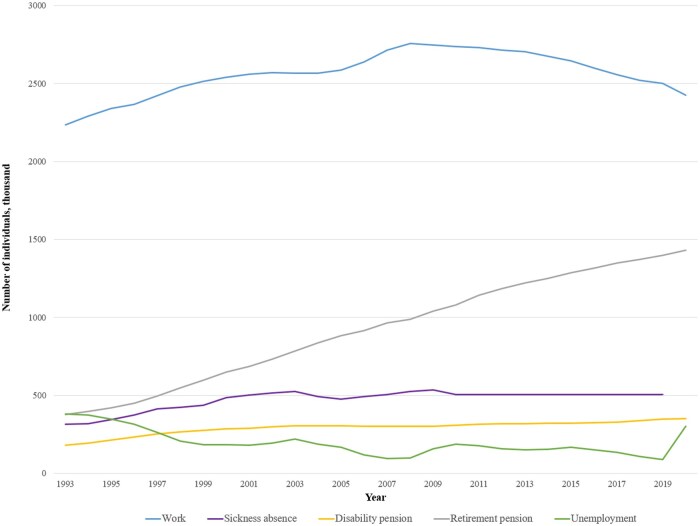
The number of individuals registered in work, sickness absence, disability pension, retirement pension, and unemployment in the period 1993–2020. Note that individuals can be registered in more than one work-related category in a given year.

Information on individuals’ temporary absences from work or other reasons for being out of employment can be obtained by utilizing information on health-related and social benefits (Table 2). Figure 3 depicts the number of individuals registered as in work or receiving various health-related and social benefits in the period 1993–2020.

## What has it found?

Using the Nor-Work cohort, gender differences in several associations between occupational exposures and withdrawal from work for older workers have been investigated [36]. Gender differences were found for associations with several occupational exposures, including high psychological job demands, monotonous work, and work with hands above shoulder height. Another study investigated gender and educational differences in work participation and working years lost to various causes [37]. Women and individuals with low education spent fewer years in work, with most years lost to health-related withdrawal from work (sickness absence and disability retirement), than men or high educated individuals, respectively. Data from Nor-Work has also been used to develop a Nordic occupational crosswalk to facilitate comparison across the country-specific occupational codes (all based on ISCO-88), before comparing prolonged sickness absence rates in specific occupational groups in the Nordic countries [38]. Out of the four countries, Norway had the highest number of occupations with relatively high rates of sickness absence compared to the general population rate, especially for musculoskeletal diseases. Manual occupational groups had the highest incidence rates overall and for musculoskeletal diseases in all four countries, whilst service occupations had the highest rates of absence due to mental issues. A recent study on occupational differences in working life expectancy and working years lost in the Nordic countries also utilizes the Nor-Work cohort [39].

Besides this, the Nor-Work cohort is currently part of a mega cohort in EPHOR, which aims to pool results from several cohorts across Europe to study occupational and non-occupational exposures and their association with non-communicable diseases [6, 12]. Nor-Work is also part of the Nordic Occupational Cancer Study, which is conducting a new follow-up study on associations between occupation and cancer in the five Nordic countries, following the previous studies in 1999 and 2009, respectively [40].

## What are the main strengths and weaknesses?

The Nor-Work cohort enables us to conduct nationwide population-based studies to better understand the relationship between individual and occupational factors, health, and labour force participation. The large amount of high-quality data and follow-up time of up to 60 years or more makes it possible to study sub-populations of interest and rare exposures or outcomes. It also allows for the construction of detailed event histories to study the complex and dynamic relationships between work-related factors, sociodemographic information, health, and labour force participation from a life-course perspective. In general, register data are collected objectively, are not subject to major bias or loss to follow-up, and have fewer issues with missing data than longitudinal cohort studies that require repeated participation [41]. Nor-Work has been, for some key variables, harmonized with large registry-based cohorts in Northern Europe, allowing for effortless pooling of cohort results through meta-analysis.

Although there is little loss to follow-up, some individuals may have missing data for certain variables due to a lack of registration in the databases, especially if they have never resided in Norway or have spent some time outside the country. An example is education, which is missing for around one-fourth of the population (Table 1). The majority of individuals with missing information on education are not Norway-born, which is to be expected, having completed formal education in their home country (or a third country) before moving to or working in Norway. However, employment data are relatively complete for the majority of individuals, as long as they have resided in Norway. Diagnoses relating to specialist treatment and cancer are relatively complete for residents of Norway, but are expected to be less complete for individuals solely working in Norway, as they often return to their home country for diagnostics and treatment [42].

Another weakness of the registry-based cohort is that it lacks both self-reported and measured information on specific occupational and non-occupational exposures (e.g. physical exposures, ambient air pollution). However, it is possible to link to JEMs to gain some of this information, albeit at the occupational level rather than the individual level. Further, the cohort does not contain information on lifestyle factors, such as smoking or leisure time physical activities, which means these factors cannot directly be accounted for in analyses. Finally, there are gaps in occupation information (between the censuses, and from 1990 to 2003), meaning it can be difficult to follow individuals’ occupation over time. Efforts have been made to impute these gaps, which mean the occupational information may be more complete than in other Norwegian registry-based cohorts. Nevertheless, imputed occupation should be used with caution, as some misclassification may be present. This misclassification will likely be greater if the complete four-digit occupational code is utilized, compared with using one to three digits only.

## Can I get hold of the data? Where can I find out more?

To use the Nor-Work cohort in research, the researcher(s) must be registered as a project member in the overarching project (working conditions, work participation, and work-related health). This registration, along with the research objectives, requires approval from the Norwegian Regional Ethics Committee (REC) South-East and the relevant registers. Researchers who are interested in collaboration/access are invited to contact Karl-Christian Nordby, cohort leader and Director of the Department of Occupational Medicine and Epidemiology at STAMI, for further information (kcn@stami.no).

## Ethics approval

Ethical approval was obtained from the Regional Committee for Medical Research Ethics—South-East Norway (REC 17344).

## Data Availability

See ‘Can I get hold of the data?’ above.
